# Supplementary material for: High-Expansion Open-Cell Polylactide Foams Prepared by Microcellular Foaming Based on Stereocomplexation Mechanism with Outstanding Oil–Water Separation
Source: Polymers (Basel). 2023 Apr 22;15(9):1984. doi: 10.3390/polym15091984 (PMC10181122; doi:10.3390/polym15091984)
Supplement: Supplementary file 1 [file polymers-15-01984-s001.zip › polymers-2314502-supplementary.pdf]

Supplementary information for

High-expansion open-cell polylactide foams prepared by microcellular foaming based on stereocomplexation mechanism with outstanding oil-water separation

Dongsheng Li, Shuang Zhang, Zezhong Zhao, ZhenYun Miao, Guangcheng Zhang*, and Xuetao Shi*

School of Chemistry and Chemical Engineering, Key Laboratory of Macromolecular Science & Technology of Shaanxi Province, Northwestern Polytechnical University, Xi'an 710072, China

S1. Characterization of synthesized 8-s-PDLA

The synthesized 8-s-PDLA by different feeding ratio were characterized by NMR, DSC and GPC. Table S1 listed the average number molecular weight and the average weight molecular weight, by both the calculation based on ^{1}H NMR spectrum and GPC measurements. This indicated the successfully synthesis of 8-s-PDLA with high productivity.

Table S1. Molecular weight of star-shaped PDLA with different feeding ratios

Feeding ratio	Theoretical molecular weight	M_n (NMR)	M_n (GPC)	M_w (GPC)	PDI	Productivity/%	T_m/°C
25:1	9500	9000	8000	10700	1.33	74	103.5
40:1	15000	13000	13200	15900	1.21	87	125.3
50:1	19000	15000	21600	23200	1.08	92	143.4
100:1	38000	39000	39200	43900	1.12	95	157.5

S2. POM photographs of the isothermal crystallization of PLLA/8-s-PDLA blends

The influence of 8-s-PDLA on the crystallization behavior of PLLA is strongly related the molecular weight of 8-s-PDLA and its added contents. Figure S2 shows the POM photographs of PLLA/8-s-PDLA blends isothermal crystallized at 130 °C. The formation of SC-PLA between 8-s-PDLA and PLLA could greatly enhance the homo-crystallization. But higher molecular weight of 8-s-PDLA-39K in Figure s1(d) would lead to high stereocomplexation density, which further confine the chain mobility. This is the explanation that addition of 8-s-PDL with higher molecular weight results in less spherulites density but bigger spherulites size.

The comparison of thermal parameters in DSC curves of pure PLLA and PLLA/8-s-PDLA is shown in Table S2.

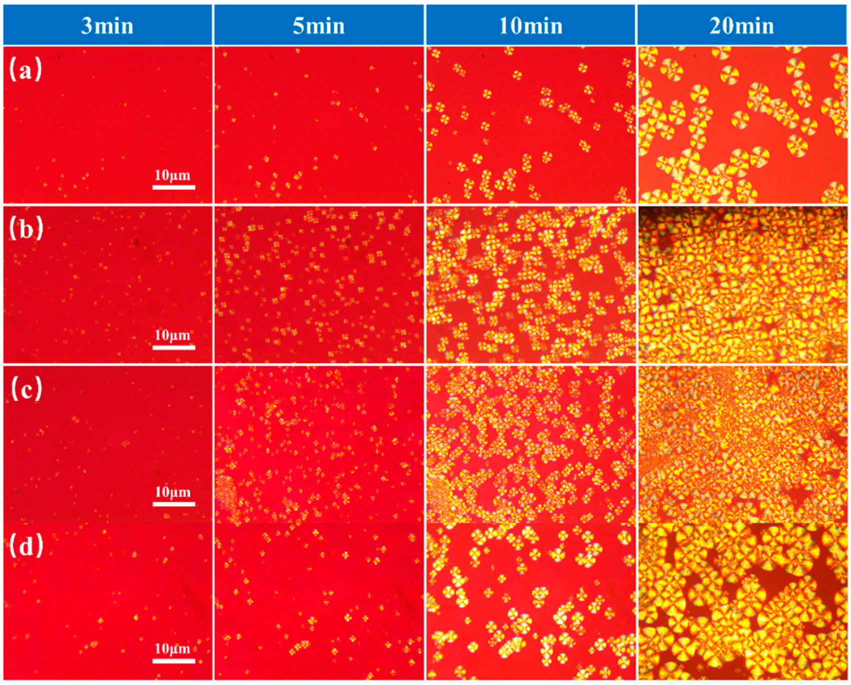

Figure S1 POM photographs of PLLA and PLLA-based blends isothermal crystallized at 130 °C: (a) pure PLLA, (b) PLLA/8-s-PDLA-13K-5%, (c)PLLA/8-s-PDLA-15K-5%, (d) PLLA/8-s-PDLA-39K-5%

Table S2. Comparison of thermal paraments of PLLA and PLLA/8-s-PDLA

Samples	T_g（°C）	T_c（°C）	$T_{m,\ PLA}$（°C）	$T_{m,\ SC\text{-}PLA}$/（°C）
PLLA	64.8	115.3	166.5	-
PLLA/8-s-PDLA-13K-3％	64.3	114.5	163.0	190.7
PLLA/8-s-PDLA-13K-5％	64.2	106.2	167.5	190.8
PLLA/8-s-PDLA-13K-10％	64.2	115.2	164.2	191.2
PLLA/8-s-PDLA-15K-5％	64.5	120.5	165.2	193.2
PLLA/8-s-PDLA-39K-5％	64.8	114.8	164.2	208.2

S3. Foaming behavior of PLLA/8-s- PDLA blends by microcellular foaming

The foaming behavior of PLLA/8-s- PDLA blends is strongly related with the foaming conditions, including the foaming temperature and foaming pressure. As shown in Figures 9 and 10 in the main text, the related characteristic parameters of PLLA/8-s-PDLA-13K foams are listed in Table S3.

Table S3. Characteristic parameters of PLLA/8-s-PDLA-13K foams prepared by different processing conditions

Foaming conditions	Cell size/(μm)	Density/ (g/cm^3)	Expansion ratio	cell density
115°C &20MPa	17.97	0.21	6.12	1.51×10^9
120°C &20MPa	22.90	0.14	8.98	1.07×10^9
125°C &20MPa	27.17	0.15	8.63	6.18×10^8
130°C &20MPa	27.95	0.15	8.61	5.67×10^8
135°C &20MPa	26.50	0.15	8.95	6.91×10^8
120°C &16MPa	25.57	0.15	8.54	7.34×10^8
120°C &22MPa	20.37	0.15	8.71	1.48×10^9
